# Cyclic nucleotide specific phosphodiesterases of *Leishmania major*

**DOI:** 10.1186/1471-2180-6-25

**Published:** 2006-03-08

**Authors:** Andrea Johner, Stefan Kunz, Markus Linder, Yasmin Shakur, Thomas Seebeck

**Affiliations:** 1 Institute for Cell Biology, University of Bern, Baltzerstrasse 4, CH-3012 Bern, Switzerland; 2 Otsuka Maryland Medicinal Laboratories, 9900 Medical Center Drive, Rockville, MD 20850, USA; 3 current address: Immunology and Infection Unit, Dept. of Biology, University of York; York YO10 5YW, UK; 4 current address: Swissmedic, Erlachstrasse 8, CH-3012 Bern, Switzerland

## Abstract

**Background:**

*Leishmania *represent a complex of important human pathogens that belong to the systematic order of the kinetoplastida. They are transmitted between their human and mammalian hosts by different bloodsucking sandfly vectors. In their hosts, the *Leishmania *undergo several differentiation steps, and their coordination and optimization crucially depend on numerous interactions between the parasites and the physiological environment presented by the fly and human hosts. Little is still known about the signalling networks involved in these functions. In an attempt to better understand the role of cyclic nucleotide signalling in *Leishmania *differentiation and host-parasite interaction, we here present an initial study on the cyclic nucleotide-specific phosphodiesterases of *Leishmania major*.

**Results:**

This paper presents the identification of three class I cyclic-nucleotide-specific phosphodiesterases (PDEs) from *L. major*, PDEs whose catalytic domains exhibit considerable sequence conservation with, among other, all eleven human PDE families. In contrast to other protozoa such as *Dictyostelium*, or fungi such as *Saccharomyces cerevisiae*, *Candida *ssp or *Neurospora*, no genes for class II PDEs were found in the *Leishmania *genomes. LmjPDEA contains a class I catalytic domain at the C-terminus of the polypeptide, with no other discernible functional domains elsewhere. LmjPDEB1 and LmjPDEB2 are coded for by closely related, tandemly linked genes on chromosome 15. Both PDEs contain two GAF domains in their N-terminal region, and their almost identical catalytic domains are located at the C-terminus of the polypeptide. LmjPDEA, LmjPDEB1 and LmjPDEB2 were further characterized by functional complementation in a PDE-deficient *S. cerevisiae *strain. All three enzymes conferred complementation, demonstrating that all three can hydrolyze cAMP. Recombinant LmjPDEB1 and LmjPDEB2 were shown to be cAMP-specific, with K_m _values in the low micromolar range. Several PDE inhibitors were found to be active against these PDEs in vitro, and to inhibit cell proliferation.

**Conclusion:**

The genome of *L. major *contains only PDE genes that are predicted to code for class I PDEs, and none for class II PDEs. This is more similar to what is found in higher eukaryotes than it is to the situation in *Dictyostelium *or the fungi that concomitantly express class I and class II PDEs. Functional complementation demonstrated that LmjPDEA, LmjPDEB1 and LmjPDEB2 are capable of hydrolyzing cAMP. In vitro studies with recombinant LmjPDEB1 and LmjPDEB2 confirmed this, and they demonstrated that both are completely cAMP-specific. Both enzymes are inhibited by several commercially available PDE inhibitors. The observation that these inhibitors also interfere with cell growth in culture indicates that inhibition of the PDEs is fatal for the cell, suggesting an important role of cAMP signalling for the maintenance of cellular integrity and proliferation.

## Background

Human pathogenic leishmanias are family of protozoa that are transmitted by female sandflies to mammalian hosts such as dogs, rodents, or humans. Three clinical forms of human leishmaniases are distinguished: visceral (caused e.g. by *Leishmania donovani*), mucocutaneous (e.g. *L. braziliensis*), and cutaneous leishmaniasis (e.g. *L. major*). All three exhibit different immunopathologies and different degrees of morbidity and mortality. Visceral leishmaniasis (Kala Azar) leads to progressive hepatosplenomegaly and is fatal if untreated. The classical mucocutaneous leishmaniasis (Espundia) leads to progressive destruction of nasal and buccal mucosa, eventually destroying nose, lips, palate and pharynx. Cutaneous leishmaniasis (oriental sore), is a localized, frequently self-healing infection of the skin at the site of the initial insect-bite, leaving ugly scars and life-long immunity [1-3]. The human leishmaniases are occurring worldwide, with endemic regions spreading and case numbers strongly increasing over the last ten years. About 12 million people are infected, with about 2 million new cases occcurring annually. Chemotherapy of the leishmaniases is in a very unsatisfactory state, and research into new drug targets and the development of novel, more effective and less toxic drugs is an urgent priority [4,5].

The *Leishmania *parasite undergoes a complex life cycle containing two different hosts, the fly vectors (*Lutzomya *spp, *Phlebotomus *ssp) and the human or mammalian hosts. Metacyclic parasites injected by the fly into the bite wound rapidly invade macrophages and differentiate morphologically and metabolically into intracellular amastigote forms. When taken up by a fly, the parasites transform in the gut of the fly into flagellated promastigote forms that eventually migrate to the salivary gland of the fly, where they differentiate again into infectious metacyclics [6]. During all these differentiation steps, the parasites are in continuous, intense contact with structures and metabolites of their hosts, requiring a repertoire of sensing and adaptation mechanisms in order to coordinate differentiation with host environment. Surprisingly little is known on the signalling processes involved in controlling and coordinating all these processes, and even less is known on the role of cAMP in this context [7,8]. Nevertheless, several independent observations indicate that cAMP is involved in the regulation of differentiation [9-11], but the mode of action of cAMP in these processes remains to be explored. With respect to the generation of the cAMP signal, adenylyl cyclases from *L. donovani *have been characterized [12]. On the other hand, PDE activity against cAMP has been demonstrated in *L. mexicana *and *L. donovani *[13], and a PDE with an unusually high K_m _for cAMP has been purified [14] from *L. mexicana*.

PDEs are ubiquitous enzymes, and they are essential for cyclic nucleotide signalling since they represent, at least for eukaryotic cells, essentially the only way for a cell to terminate a cyclic nucleotide signal. In addition, the PDEs are largely responsible for confining a cyclic nucleotide signal to particular locations, and to prevent its diffusion throughout the cell [15-17]. Three classes of PDEs have been identified based on their different catalytic domains. Class I [18] is found in almost all eukaryotes, and it represents the only PDE class of higher eukaryotes. Class I PDEs have also been identified in the kinetoplastid protozoa *Trypanosoma brucei*, the causative agent of African human sleeping sickness [19-21] and *Trypanosoma cruzi*, the causative agent of the South American Chagas disease [22-24]. Class II PDEs [25] are found in some prokaryotes (e.g. *Vibrio fischeri*) and in many lower eukaryotes such as *Dictyostelium discoideum *or fungi such as *Saccharomyce*s, *Candida *or *Neurospora. Dictyostelium *and fungi contain genes for both, class I and class II PDEs. A special case is represented by *Schizosaccharomyces pombe *that contains only a single, class II PDE gene. The completely unrelated class III PDEs [26] are confined to the prokaryotes.

The human genome codes for eleven PDE families, most of which contain several family members. In addition to this genetic variety, numerous splice variants are generated from many of the genes, resulting in a PDE proteome of about sixty PDE isoenzymes. The catalytic domains of all PDE family members share considerable homology in their amino acid sequences, and their three-dimensional structures appear to be closely similar. Despite the overall similarity of their catalytic domains, the individual PDEs exhibit characteristic selectivities for cAMP and/or cGMP [27] as their substrates, and each displays a unique inhibitor specificity profile.

Besides their interest for basic cell biology, the human class I PDEs have become interesting targets for drug development. Sub-type and tissue-specific PDE inhibitors constitute a growing class of pharmaceutical compounds that find applications for a broad spectrum of maladies [15,28,29].

The recent completion of a number of kinetoplastid genomes has allowed to gain an overview over the PDE repertoire in these organisms. We here present the identification of the five class I PDEs from *Leishmania*, and report an initial characterization for three of them.

## Results

### Identification of five putative cyclic nucleotide specific PDEs of *L. major*

**Figure 1:**
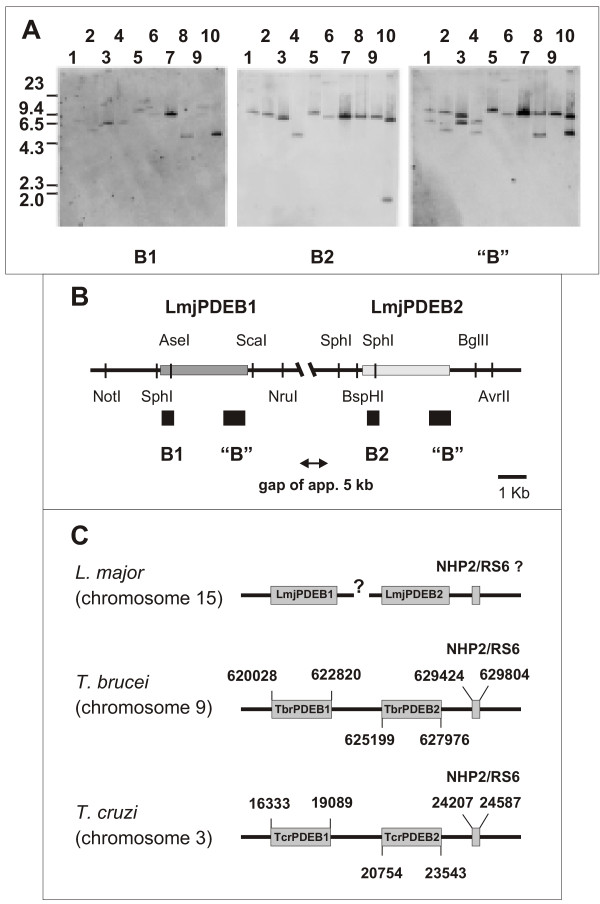
Genomic organization of PDE genes. Panel A: Southern blot analysis. Genomic DNA of *L. major *was digested with BspHI (lane 1), BspHI plus ScaI (lane 2), BspHI plus NruI (lane 3), BspHI plus AvrII (lane 4), NotI (lane 5), NotI plus BglII (lane 6), NotI plus AvrII (lane 7), NotI plus ScaI (lane 8), AseI (lane 9) and SphI (lane 10). The blots was hybridized with probes B1 and B2 that specifically recognize *LmjPDEB1 *or *LmjPDEB2*, respectively (see panel B), or with probe "B" that hybridizes to both genes. The sizes of the DNA size markers are given on the left. Panel B: Organization of the LmPDEB locus on chromosome 15. Restriction sites used in the mapping are indicated. The uncharacterized stretch between the two coding regions is estimated to be about 5 kb long. Filled boxes labelled B1, B2 and "B" indicate the gene regions used as hybridization probes. Panel C: Genomic organization of the *LmjPDEB1/LmjPDEB2 *locus on chromosome 15 of *L. major*. The organization of the corresponding loci of *T. brucei *and *T. cruzi *are given for comparison. T. brucei: *TbrPDEB1 (old designation: TbPDE2C; *Tb09.160.3590) and *TbrPBEB2 (old designation TbPDE2B; *Tb09.160.3630) and NHP/2RS6 (Tb09.160.3670). *T. cruzi*: TcrPDEB1 (Tc00.1047053508277.100) followed by TcrPDEB2 (old designation TbPDE1; Tc00.104053508277.120; GenBank accession number AAP49573) and by NHP2/RS6 (Tc00.104053508277.120). Numbers indicated nucleotide positions on the respective chromosomes.

Screening the *Leishmania major *genome database [30] with amino acid sequences of class I PDE catalytic domains identified five genes that are predicted to code for class I PDEs (Fig. 1). Chromosome 18 contains the single copy gene *LmjPDEA *(GeneDB identification number: LmjF18.1090) that is predicted to code for a polypeptide of 631 amino acids. Its catalytic domain comprises amino acids 384 – 609 (as defined by the conserved domain database), with no other functional domains discernible. The open reading frame of LmjPDEA was amplified from genomic DNA and sequenced and its sequence was submitted to GenBank under the accession number AY462262.

Chromosome 15 contains a locus containing two tandemly arranged PDE genes, *LmjPDEB1 *and *LmjPDEB2 *(Fig 1). The open reading frames of both genes were amplified from genomic DNA and were sequenced and the sequences have been submitted to GenBank under the following accession numbers: *LmjPDEB1*: AY462264, and *LmjPDEB2*: AY462263.

The sequencing data, in conjunction with Southern blot and PCR analyses demonstrated that the current version of the *L. major *database (version of July 15, 2005) contains an assembly error in this region, in that it represents only a single PDE gene that is a chimera between *LmjPDEB1 *and *LmjPDEB2*. The genomic organization of the LmjPDEB locus now presented in Fig 1B closely corresponds to that found for the homologous PDE genes in the *T. brucei *and the *T. cruzi *genomes. In *T. brucei*, *TbrPDEB1 *(old designation: *TbPDE2C; *Tb09.160.3590) and *TbrPDEB2 (*old designation: *TbPDE2B; *Tb09.160.3630) are also tandemly arranged on chromosome 9, with 2379 bp between the two open reading frames, followed by a gene for the small non-histone protein NHP2/RS6 (Fig 1C). In *T. cruzi*, the two homologues are similarly arranged on chromosome 3, in the sequence *TcrPDEB1 *(Tc00.1047053508277.100) followed by *TcrPDEB2 *(old designation *TcPDE1*; GenBank accession number AAP49573; [24]) and by *NHP2/RS6*.

### *LmjPDEB1 *and *LmjPDEB2 *produce stable transcripts

**Figure 2:**
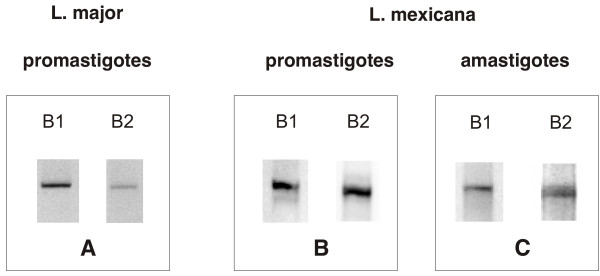
Northern blots. Total RNA from *L. major *promastigotes (panelA), *L. mexicana *promastigotes (panel B) and *L. mexicana *amastigotes (panel C) was fractionated on agarose gels, and the RNA-filters were hybridized with DIG-labelled DNA probes for *LmjPDEB1 *(B1) and LmjPDEB2 (B2). The sizes of the two mRNAs were 5.3 Kb and 4.9 Kb for B1 and B2, respectively.

**Figure 3:**
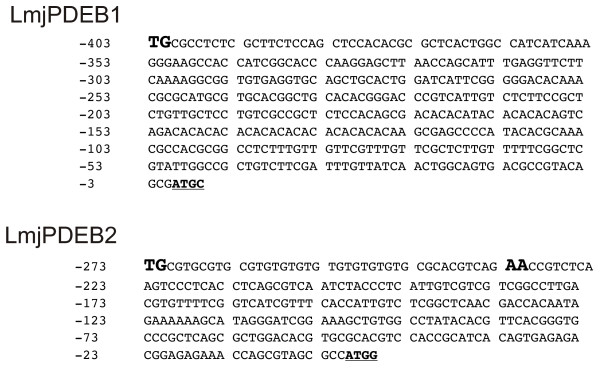
5'-UTR sequences of *LmjPDEB1 *and *LmjPDEB2*. Larger, bold letter indicate the various splice acceptor sites. Bold, underlined letters symbolize the start of the open reading frames.

Expression of the two genes in *L. major *promastigotes was analyzed by Northern blot analysis and by RT-PCR. When Northern blots of total RNA were hybridized with the respective probes, stable mRNA was detected for both of them (Fig. 2, panel A). Similar results were also obtained using total RNA of *L. mexicana *promastigotes and amastigotes (Fig. 2, panels B and C), suggesting that the two genes are similarly expressed in both life cycle stages. The 5'-termini of the respective mRNAs in *L. major *promastigotes were analyzed by RT-PCR amplification using a common, mini-exon specific forward primer and individual gene-specific reverse primers for each gene. For *LmjPDEB1*, a single type of mRNA was found, with an unusually long 5'-UTR region of 403 nucleotides. For *LmjPDB2*, two splice variants were identified, with 5'-UTR regions of 273 and 233 nucleotides, respectively (Fig. 3).

### Amino acid sequence analysis of LmjPDEA, LmjPDEB1 and LmjPDEB2

The polypeptide chain of LmjPDEA consists of 631 amino acids without any recognizable functional domain, except for the class I PDE catalytic domain at the C-terminus (amino acids 384 – 609).

**Figure 4:**
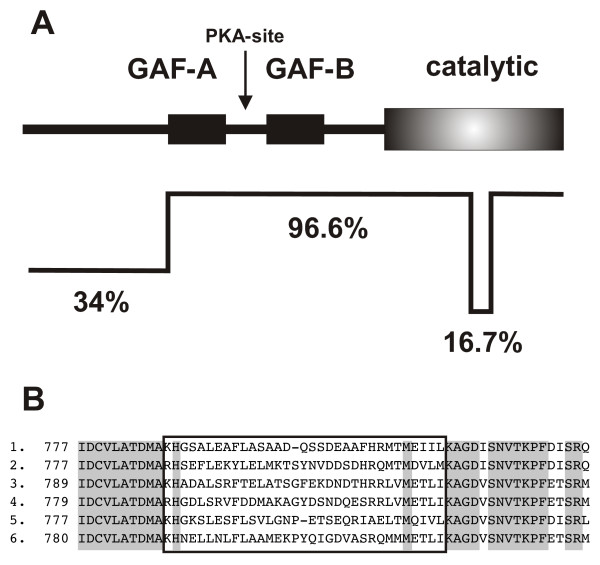
Sequence organization and comparison of LmjPDEB1 and LmjPDEB2. Panel A: Extent of amino acid sequence identity between various parts of LmjPBEB1 and LmjPDEB2. A 24 amino acid stretch within the otherwise identical catalytic region is entirely dissimilar between the two. Panel B: similar stretches of sequence divergence (boxed area) are found at identical positions within the otherwise highly conserved catalytic regions the *T. brucei *and *T. cruzi *homologues. 1: TbrPDEB1; 2: TbrPDEB2; 3: LmjPDEB1; 4: LmjPDEB2; 5: TcrPDEB1; 6: TcrPBEB2.

The open reading frames of *LmjPDEB1 *(2820 bp) and *LmjPDEB2 *(2790 bp) code for proteins of 940 and 930 amino acids, respectively. The two share an overall 82 % sequence identity, with a markedly uneven distribution along the polypeptides. The first 230 amino acids are highly diverged (only 34 % amino acid sequence identity), whereas the remainder of the sequence is almost completely conserved (96.6 % sequence identity). The only exception is a stretch of 24 amino acids in the catalytic domain that are entirely dissimilar between LmjPDEB1 and LmjPDEB2 (Fig. 4). Interestingly, similar stretches of divergence within the catalytic domains are also found, at identical positions, in the respective homologues of *T. brucei *and *T. cruzi*. LmjPDEB1 and LmjPDEB2 both contain two closely spaced GAF domains [31] in their N-terminal regions (LmjPDEB1: GAF-A amino acids 250-398, GAF-B 422 – 567; LmjPDEB2: GAF-A: 240 – 388, GAF-B: 412 -557). Between the two GAF domains, a putative protein kinase A phosphorylation site is located (KKKS; LmjPDEB1 402 – 404; LmjPDEB2 392 – 395). This is the only protein kinase A site that is also conserved, and found at an identical position, in the *T. brucei *and *T. cruzi *homologues.

The catalytic domains of the leishmanial PDEs are highly conserved, and they all contain the signature sequence [HD(LIVMFY)XHX(AG)XXNX(LIVMFY)] that characterizes them as class I PDEs [18]. The amino acid sequence identity between the catalytic domains of LmjPDEB1 and LmjPDEB2 and the corresponding regions of the human PDEs 1 – 11 varies between 41.7% (to HsPDE3A) to 48.9% (to HsPDE10A). Based on the publicly available 3D-structures of several human PDE catalytic domains [32], sixteen amino acid residues are absolutely conserved between all human PDEs (Table 1). In only two of these positions, some of the leishmanial PDEs exhibit amino acid replacements, both in positions that are not directly located in the active site. A conserved alanine residue (A^466 ^in HsPDE4B) is replaced by G^741^, G^731 ^and Y^673 ^in LmjPDEB1, LmjPDEB2 and LmjPDEC, respectively. In addition, a conserved histidine residue (H^478 ^in HsPDE4B) is replaced by leucine (L^459 ^in LmjPDEA) or methionine (M^685 ^in LmjPDEC). Of the two residues that confer selectivity for cAMP over cGMP, Q^443 ^and N^567 ^in HsPDE4B [33], the glutamine residue is conserved in all leishmanial PDEs. In the position corresponding to N^567^, an asparagine residue is maintained in all leishmanial PDEs except for LmjPDEC. Here the corresponding position is taken by an alanine residue (A^770 ^in LmjPDEC). This suggests that LmjPDEC might represent a dual-substrate PDE, as has already been experimentally demonstrated for its *T. cruzi *homologue, TcrPDEC [22]. While the catalytic domains of all five *L. major *PDEs share a high degree of similarity, the two catalytic domains of LmjPDEB1 and LmjPDEB2 are completely identical, with the exception of a stretch of 24 amino acids that comprises the predicted helices 12 and 13. Similar non-conserved stretches of comparable length and at identical locations are also observed in the homologues of *T. brucei *and *T. cruzi *(Fig 4).

### Functional complementation of a PDE-deficient *S. cerevisiae *strain by LmjPDEA, LmjPDEB1 and LmjPDEB2

**Figure 5:**
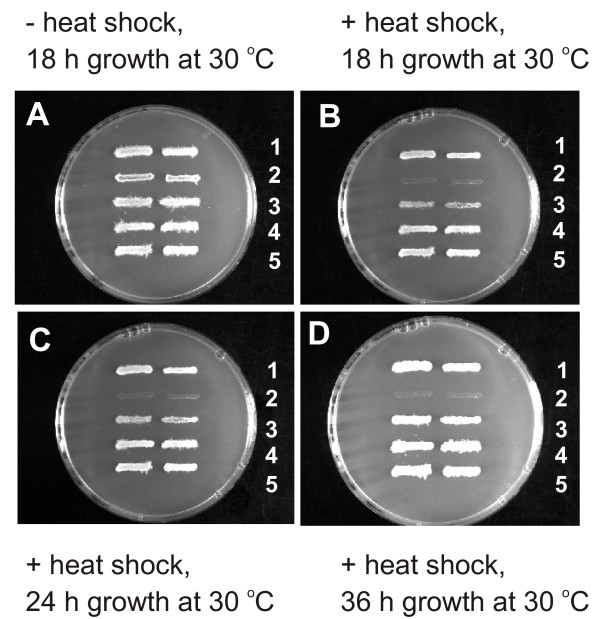
Complementation of the PDE-deficient *S. cerevisiae *strain PP5. Panel A: plate without heat-shock treatment, incubated at 30°C for 18 h; panels B – D: plates heat-shocked for 15 min at 55°C, and subsequently incubated at 30°C for 18 h (panel B), 24 h (panel C) or 36 h (panel D). The plates always display rows of patches of two independent transformants transfected with the following plasmids: Row 1: pLT1 + *TbrPDEA *(= positive control); row 2: pLT1, empty plasmid (= negative control); row 3: pLT1 + *LmjPDEA*; row 4: pLT1 + *LmjPDEB2*; row 5: pLT1 + *LmjPDEB1*.

Deletion of the two PDE genes *ScPDE1 *and *ScPDE2 *from the *S. cerevisiae *genome renders the mutants highly sensitive to stress conditions such as a heat-shock. Heterologous complementation of this heat-shock phenotype has proven to be a highly sensitive validation procedure for suspected PDE genes [19,34]. The full-size open reading frames of LmjPDEA, LmjPDEB1 and LmjPDEB2 were cloned into the yeast expression vector pLT1 [19] and expressed in the PDE-deficient *S. cerevisiae *strain PP5 [35]. Transformants were patched and tested for heat shock resistance (Fig 5). All three PDE genes restored heat shock resistance to the indicator strain, though with different efficiencies. *LmjPDEB1 *and *LmjPDEB2 *completely restored the wild-type phenotype, whereas complementation by *LmjPDEA *was much weaker, as seen at short incubation times after the heat shock. Patches expressing LmjPDEA had only grown weakly when observed after 18 h post-heat-shock incubation at 30°C (Fig. 5B), while patches expressing LmjPDEB1 or LmjPDEB2 already showed vigorous growth after this time. When observed after 36 h of incubation, all three recombinant strains had grown to a similar extent (Fig. 5D). The results of these complementation experiments established that all three genes code for functional PDEs, and they demonstrate that these PDEs can use cAMP as their substrate.

### Catalytic activity

**Figure 6:**
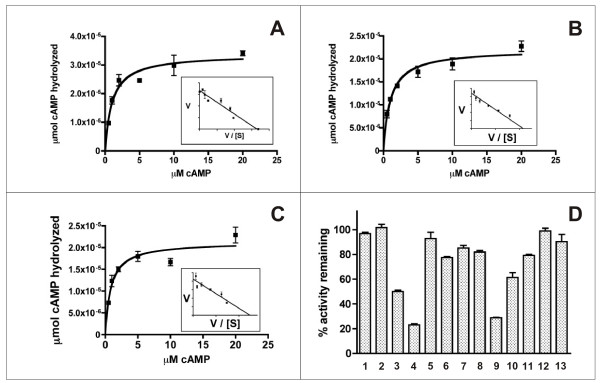
Characterization of PDE activity and inhibitor potency. Panel A: Michaelis-Menten kinetics of recombinant LmjPDEB2 with ^3^H-cAMP as substrate. Panel B: reaction in the presence of a 100-fold excess of unlabelled cGMP. Panel C: reaction in the presence of a 50-fold excess of unlabelled AMP. Inserts in A – C: corresponding Eadie-Hofstee plots. Panel D: Effect of PDE inhibitors on LmjPDEB2 activity (1 μM ^3^H-cAMP substrate, 100 μM inhibitor). 1, cilostamide; 2, zaprinast; 3, etazolate; 4, dipyridamole; 5, Ro-20-1724; 6, rolipram; 7, IBMX; 8, 8-methoxy-methyl-IBMX; 9, trequinsin; 10, papaverine; 11, milrinone; 12, pentoxifylline; 13, erythro-9-(2-hydroxy-3-nonyl)adenine (EHNA). Very similar results were obtained with LmjPDEB1.

Soluble cell lysates were prepared from yeast strains expressing each of the three PDEs and were assayed for PDE activity. Lysates prepared from the LmjPDEA expressing strain consistently showed no measurable PDE activity. While the reason for this is still unclear and might reflect a trivial technical problem, the observation is in line with the finding that *LmjPDEA *complements the PDE-deficient yeast strain PP5 much less efficiently than do the two other PDE genes (see Fig. 5). In contrast to *LmjPDEA*, lysates from yeast strains expressing *LmjPDEB1 *and *LmjPDEB2 *showed strong PDE activities. The two enzymes exhibit K_m _values of 1 and 7 μM for cAMP, respectively, well within the range of other class I PDEs (Table 2). The apparent K_m _for cAMP (0.97 ± 0.09 μM) was not altered by the presence of a 100-fold excess of cGMP (Fig 6A and 6B), nor was V_max _(2.56 ± 0.71 nmol/mg lysate/15 min), indicating that LmjPDEB1 and LmjPDEB2 are specific for cAMP, and that their activity is not modulated by cGMP. A 50-fold excess of the reaction product 5'-AMP also did not alter the K_m _(Fig 6C), indicating that the PDEs are not subject to marked product inhibition. For all parameters determined, LmjPDEB1 and LmjPDEB2 behaved very similarly, indicating that the small stretch of sequence divergence between the two catalytic domains does not markedly alter their functionality. Also, the divergent N-terminal regions do not seem to affect the kinetics under in vitro conditions.

### Inhibitor profiling

As an initial survey, a number of commercially available inhibitors that are frequently used in cell biological studies were tested at a 100-fold excess over substrate (100 μM inhibitor vs 1 μM cAMP; Fig. 6D), using extracts from yeast cells expressing the recombinant PDEs. Most of the inhibitors tested (cilostamide, zaprinast, etazolate, dipyridamole, Ro-20-1724, rolipram, isobutyl-methylxanthine (IBMX), 8-methoxymethyl-IBMX, trequinsin, papaverine, milrinone, pentoxifylline, and erythro-9-(2-hydroxy-3-nonyl)adenine (EHNA) were essentially inactive, even at the high concentration used in these experiments. Only dipyridamole (IC_50 _= 29 μM), trequinsin (IC_50 _= 96 μM) and etazolate caused significant inhibition.

### Inhibition of cell proliferation

**Figure 7:**
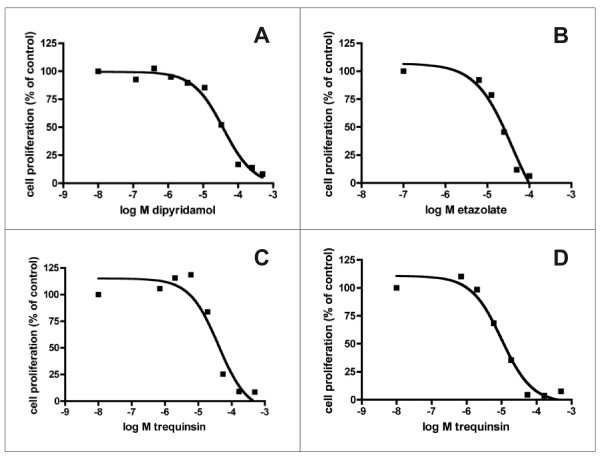
Inhibition of cell proliferation in culture. The EC_50 _values for *L. major *promastigote proliferation were determined for dipyridamole (panel A), etazolate (panel B) and trequinsin (panel C). Panel D represents an EC_50 _determination for trequinsin with *L. infantum *amastigotes. EC_50 _values were determined various time points and cell densities, and all growth experiments were repeated at least three times. Representative growth curves for each inhibitor are shown. EC_50 _values for all inhibitors were in the 30 – 100 μM range for both promastigotes and amastigotes.

Dipyridamole, etazolate and trequinsin were further tested for their effect on cell proliferation (Fig. 7). Promastigote cultures of *L. major *or amastigote cultures of *L. infantum *were grown in the presence of various concentrations of inhibitors, or in the presence of 1 % (v/v) dimethylsulfoxide as a control. All three compounds were strongly inhibitory (IC_50 dipyridamole _= 44.7 ± 12.2 μM; IC_50 etazolate _= 57.5 ± 27.0 μM; IC_50 trequinsin _= 43.6 ± 3.7 μM for promastigotes; and IC_50 trequinsin _= 10.2 ± 4.2 μM for amastigotes, respectively). All three inhibitors are markedly more potent than the frequently used, wide-spectrum PDE-inhibitor isobutyl-methyl-xanthine (1.03 ± 0.67 mM). The inhibitory effects were independent of cell density and were not reduced upon prolonged incubation of the culturesThese observations indicate that the inhibitors are metabolically stable, and that the PDEs may be of similar importance for proliferation of both promastigotes and amastigotes in culture.

## Discussion

The current study reports on the identification and characterization of three class I cyclic nucleotide-specific PDEs from *L. major*. LmjPDEA is a single copy gene, while the two genes for LmjPDEB1 and LmjPDEB2 are tandemly arranged on chromosome 15 and code for two similar enzymes. Very similar enzymes also have been identified in T. brucei and other kinetoplastids, and a unifying nomenclature for kinetoplastid PDEs has recently been proposed [36]. The catalytic domains of the leishmanial PDEs are very similar to those of the human PDEs, and all functionally important amino acids are conserved.

*LmjPDEA*, *LmjPDEB1 *and *LmjPDEB2 *complement the PDE-defective *S. cerevisiae *strain PP5 and restore its wild-type heat-shock resistance phenotype. However, complementation by *LmjPDEA *was less effective (slower cell growth after heat shock) than that by the other two (see below). When yeast cell lysates were prepared from *LmjPDEA*-complemented strains, no PDE activity was detectable. This is similar to what was observed earlier with the highly conserved *T. brucei *homologue of *LmjPDEA*. This gene, *TbrPDEA*, also complements the PP5 strain, but only very little enzyme activity was detectable in yeast cell lysates [19]. This lack of biochemically detectable activity despite functional complementation may reflect some specific property common to the LmjPDEA and TbrPDEA enzymes.

LmjPDEB1 and LmjPDEB2 contain catalytic domains that are identical, except for a 24 amino acid sequence that is highly diverged between the two. This sequence spans the region of predicted helices 12 and 13 [32]. Divergent regions of similar length are also found at the corresponding locations of the homologous PDEs of *T. brucei *and *T. cruzi *(see below). These regions might confer subtle functional or regulatory specificities to the otherwise highly conserved catalytic domains of the respective isoenzymes of each species.

For *LmjPDEB1 *and *LmjPDEB2 *highly conserved, homologues genes exist also in other kinetoplastid protozoa, *T. brucei *and *T. cruzi*. In both organisms, the two homologues *TbrPDEB1 *(old nomenclature: TbPDE2C [20]) and *TbrPDEB2 *(old nomenclature: TbPDE2B [21]) on chromosome 9 of *T. brucei*, and *TcrPDEB1 *and *TcrPDEB2 *(old nomenclature: TcPDE1 [24]) on chromosome 3 of *T. cruzi *are tandemly arranged with the two open reading frames spaced by about 2500 bp, and followed by an open reading frame of an *NHP2/RS56 *gene coding for a non-histone protein. Our results suggest that *LmjPDEB1 *and *LmjPDEB2 *are similarly arranged on chromosome 15 of *L. major*.

*LmjPDEB1 *and *LmjPDEB2 *both complement the PDE-deficient yeast strain PP5 very effectively, and high enzyme activities were present in the respective yeast cell lysates. Both enzymes behave very similarly, exhibiting K_m_s in the low μM range for cAMP as a substrate, and they are entirely specific for cAMP. When a number of PDE inhibitors were tested on recombinant LmjPDEB1 and LmjPDEB2, most of them exhibited very low potency. This marked insensitivity of leishmanial PDEs to many PDE inhibitors, including the broad-spectrum inhibitor IBMX, corresponds to what was found with the *T. brucei *homologues. Only dipyridamole, trequinsin and etazolate exhibited at least a moderate potency.

The three inhibitors also inhibited proliferation of *L. major *promastigotes in culture, all with IC_50 _values in the 30 – 100 μM range. Very similar EC_50 _values are also obtained when the same compounds were used with *L. infantum *amastigotes, indicating that both life cycle stages are similarly sensitive to these inhibitors. Though inhibitor specificity cannot be taken for granted at these relatively high concentrations, the experiments clearly suggest that the PDEs may be essential enzymes whose inhibition blocks cell proliferation.

## Conclusion

The identification and molecular characterization of the leishmanial PDEs will now allow a more detailed investigation of the role of cAMP signalling in *Leishmania *biology. On the other hand, these PDEs may represent novel drug targets. They belong to a family of enzymes that has already been successfully exploited by medicinal chemistry for a number of human disease conditions. The range of applications for PDE inhibitors might yet be extended further to include antiprotozoal drugs.

## Methods

### Materials

^3^H-labeled cAMP was purchased from Hartmann Analytics, Braunschweig, Germany (Cat. Nr MT 616, 15-50 Ci/mmol). After purification by ion-exchange chromatography on QAE-Sephadex A25, it was stored frozen at -20°C. Oligonucleotide primers were purchased either from Microsynth, Balgach, Switzerland, or from Qiagen. DNA sequencing was done using the ABI Prism BigDye Terminator v3.0 Cycle Sequencing Ready Reaction Kit (Applied Biosystems). Sequences were analyzed on an ABI 3100 instrument at the Computational and Molecular Population Genetics Lab, University of Bern.

### Cell culture

*Leishmania major *MHRO/IR/75/ER or LV39 promastigote forms were cultivated at 27°C in SDM medium containing 5% heat-inactivated foetal bovine serum [37]. The MHRO/IR/75/ER isolate was originally recovered from a patient in Iran, and was obtained through Nicolas Fasel, Dept. of Biochemistry, University of Lausanne. Cell proliferation assays were done in 5 ml cultures to which drugs dissolved in DMSO were added. The final DMSO concentration was 1 % in all cases, and appropriate DMSO controls were always included. At various times, 150 ml aliquots were withdrawn and absorbance was measured at 600 nm in a microtiter plate reader. The correlation between OD_600 _and cell number was strictly linear over at least the range between 3 × 10^5 ^and 4 × 10^7 ^cells/ml.

### Identification and cloning of PDE genes

The *Leishmania *genome database [30] was searched with the amino acid sequences of the cAMP-specific PDEs from *Trypanosoma brucei *TbrPDEA (old nomenclature: TbPDE1; GenBank accession number AAL58095) and TbrPDEB1 (old nomenclature: TbPDE2C; GenBank accession number AAK33016). The genes to be characterized in this study were named LmjPDEA, LmjPDEB1and LmjPDEB2, in accordance with the recently proposed unified nomenclature [36]. Based on the database sequences, PCR primers were designed to amplify the open reading frames of LmjPDEA, LmjPDEB1 and LmjPDEB2. Forward primers for *LmjPDEA*, *LmjPDEB1 *and *LmjPDEB2*, respectively were LmPDEAup1 (5'-GTGGTCGA**CTCGACTTTCTTGAGCAG**-3'), LmPDEB2up1 (5'-GATGTCGA**CATTCAGCGGTCTTTTCC**-3') and LmPDEBup1 (5'-GATGTCGACTG**GCATATTTCACGGCCA**-3'). All contained a *Sal*I site (underlined) followed by the respective gene sequence without the start codon (boldface type). The reverse primers for *LmjPDEA*, *LmjPDEB1 *and *LmjPDEB2*, respectively were LmPDEAlo1 (5'-CTGGGAATCCTA*AGCATAATCTGGAACATCATATGGATA*C**GAGTCGTCGTGGTTGG**-3'), LmPDEB2lo1 (5'-CTGGGAATCCTA*AGCATAATCTGGAACATCATATGGATA***AACAATCGAGGATCGGATG**-3') and LmPDEBlo1 (5'-CTGGGAATC CTA*AGCATAATCTGGAACATCATATGGATA***AACAATCGAGGGTCGGATG**-3'). . All reverse primers contained a stop codon (underlined) and a preceding sequence coding for a hemagglutinin tag (-YPYDVPDYA; in italics), followed by the gene-specific sequence (boldface). PCR products with sizes of 1.9 kbp (*LmjPDEA*) and 2.8 kbp (*LmjPDEB1 *or *LmjPDEB2*) were cloned into the pCR2.1 vector (Invitrogen) and were verified by sequencing.

### Southern blot analysis

Genomic DNA was isolated from *L. major *promastigotes, digested with the appropriate restriction enzymes, separated on 0.8 % agarose gels and transferred to a positively charged nylon membrane (Roche). Digoxigenin-labelled DNA probes were generated using the PCR DIG probe synthesis kit (Roche). Probes were amplified with the following primers: LmPDEAup5 (5'-TAACCACCCGAAGGAGTACG-3') and LmPDEAlo5 (5'-CTCGGCTACCTGAGAGTTGG-3'), resulting in a fragment of 449 bp specific for *LmjPDEA*; LmPDEB2up6 (5'-CGTCGGACTGGTACATCCTT-3') and LmPDEB2lo6 (5'-GTTTGCGATCACCATGTACG-3') produced a 312 bp fragment specific for *LmjPDEB1*; LmPDEBup5 (5'-CTGCATTCTGAGCCGTTACA-3') and LmPDEBlo5 (5'-AATGGTAACGGTCGTCTTCG-3') produced a 394 bp fragment specific for *LmjPDEB2*. A hybridization probe recognizing both genes, *LmjPDEB1 *and *LmjPDEB2*, was amplified by PCR with the primers LmPDEBup6 (5'-CCTGCAGCGTAACAGCATTA-3') and LmPDEBlo6 (5'-GCGAGTCCGTCTTCAGGTAG-3'; fragment length 476 bp). In order to achieve a minimal hybridization background, the DNA templates for the PCR reactions were excised from the respective plasmid vectors by digestion with *Bam*HI and *Sal*I and were purified by gel extraction (QIA quick Gel Extraction Kit, Qiagen). Blots were prehybridized for 6 h in DIG Easy Hyb buffer (Roche) and were hybridized at 42°C overnight in the same buffer containing 20 ng/ml of DIG-labelled probe. High stringency washes were done in 0.5 × SSC, 0.1 % SDS at 68°C twice for 15 min. Hybridization signals were detected with an alkaline phosphatase-conjugated anti-DIG antibody (Roche) and the CDP-Star substrate (Roche) and were visualized on a LAS-1000 Image Reader (Fuji).

### Northern blot analysis

Total RNA from *L. major *promastigotes or *L. mexicana *amastigotes was denatured in 50 % (v/v) DMSO, 4 % (v/v) deionised glyoxal and 10 mM sodium phosphate, pH 6.85, for 5 min at 50°C and separated on a 1.2 % agarose gel in 10 mM sodium phosphate. RNA was transferred to positively charged nylon membranes (Roche) by capillary force. Prehybridization and hybridization with the DIG-labelled probes were done as described above, but at a hybridisation temperature of 50°C. High stringency washes and hybridisation signal detection were done as described above. A hybridization probe specific for α-tubulin was used to normalize all blots.

### Expression in *S. cerevisiae*

The leishmanial PDEs were expressed in the PDE-deficient *S. cerevisiae *strain PP5 (*MATa leu2-3 leu2-112 ura3-52 his3-532 his4 cam pde1::ura3 pde2::HIS3*), a gift of John Colicelli (UCLA). The hemagglutinin-tagged, full-length PDE genes were excised from the cloning vectors by digestion with *Bam*HI and *Sal*I, purified by gel extraction and introduced into the yeast expression vector pLT1 [19]. The pLT1 vector contains a strong TEF 2 promotor, followed by an optimized Kozak sequence, the start codon and a *Sal*I site (5'-*CTAAAC***ATG**GTCGAC-3'; Kozak sequence in italics, start codon in boldface and *Sal*I site underlined). Transformation into the yeast strain PP5 was done exactly as described [19].

### Complementation assay

The heat-shock assay to detect complementation of the PDE-deficient phenotype of the *S. cerevisiae *strain PP5 was done exactly as described [19]. Single yeast colonies were patched onto YPD plates prewarmed to 55°C, and were incubated for another 15 min at 55°C. Plates were then cooled to room temperature and were incubated at 30°C for 18 – 36 h.

### Yeast cell lysis

Yeast cell lysis was performed as described by Kunz et al [19] with minor modifications. Briefly, yeast cells grown to mid-log to end-log phase in SC-leu medium were collected, resuspended in the original volume of prewarmed YPD medium, and incubated for an additional 3.5 h at 30°C to maximize protein expression. Cells were then harvested and washed twice in H_2_O, and the washed cell pellet was stored overnight at -70°C. For preparing the lysate, the cells were thawed on ice and suspended in ice-cold extraction buffer (50 mM Hepes pH 7.5, 100 mM NaCl, 1 × Complete^® ^protease inhibitor cocktail without EDTA (Roche)). Cells were lysed by grinding with glass beads (0.45 – 0.50 mm) in 2 ml Sarstedt tubes, using a FastPrep FP120 cell disruptor (3 × 45 s at setting 4). The subsequent centrifugation steps were done exactly as described. To the resulting supernatant, glycerol was added to a final concentration of 15 % (v/v), aliquots were snap-frozen in liquid nitrogen and were stored at -70°C.

### Phosphodiesterase assay

PDE activity was determined in 50 mM HEPES, pH 7.5, 0.5 mM EDTA, 10 mM MgCl_2_, 50 mg/ml BSA in a final assay volume of 100 μl. Each assay contained 50'000 cpm ^3^H-labelled cAMP, with unlabeled cAMP added to adjust the desired total substrate concentration. Reactions were run at 30°C and were linear for at least 60 min. The standard reaction time was set to 15 min, and the amount of enzyme was always chosen so that no more than 15 % of the substrate was hydrolyzed. Inhibitor studies were done at a cAMP concentration of 1 μM. Inhibitors were dissolved in DMSO, but the final DMSO concentration in the assays never exceeded 1%. Control reactions with DMSO alone were always included. Reactions were stopped by the addition of 25 μl of 0.5 N HCl. For the subsequent dephosphorylation of the AMP, the stopped reactions were neutralized with 20 μl 1 M Tris base, followed by the addition of 10 μl of calf intestinal alkaline phosphatase (Roche Diagnostics; 1 unit/10 μl). The dephosphorylation reactions were incubated for 15 min at 37°C and were then applied to 1 ml columns of QAE-Sephadex A25 in 30 mM ammonium formiate, pH 6.0. The ^3^H-adenosine formed during the reaction was eluted with 1.6 ml of 30 mM ammonium formiate, pH 6.0 and was collected into 3.5 ml water-miscible scintillation fluid (Packard Ultima Flo). Assays were always run in triplicates, and at least three independent experiments were performed in every case. Data were analyzed using the GraphPad Prism software package.

## Authors' contributions

AJ and SK carried out the molecular and genetic experiments, ML was measuring enzyme activities, TS performed the cell culture studies, and YS and TS conceived the study and drafted the manuscript. All authors read and approved the final manuscript.

## Figures and Tables

**Table 1:** Amino acid replacements of conserved residues in the catalytic domains of *L. major *PDEs. Amino acid conservation is indicated by a dash.

HsPDE4B	Y^399^	**H**^406^	**N**^407^	**H**^410^	**H**^446^	**D**^447^	**H**_450_	G^452^	A^466^	E^476^	H^478^	**H**^479^	**T**^517^	**D**^564^	**E**^585^	**Q**^615^
LmjPDEA	-	-	-	-	-	-	-	-	-	-	L^459^	-	-	-	-	-
LmjPDEB1	-	-	-	-	-	-	-	-	G^741^	-	-	-	-	-	-	-
LmjPDEB2	-	-	-	-	-	-	-	-	G^731^	-	-	-	-	-	-	-
LmjPDEC	-	-	-	-	-	-	-	-	Y^673^	-	M^685^	-	-	-	-	-
LmjPDED	-	-	-	-	-	-	-	-	-	-	-	-	-	-	-	-

**Table 2:** K_m _of PDEs

**PDE**	**cAMP (μM)**	**cGMP (μM)**	Refs
LmPDEB1	0.99	---	*)
LmPDEB2	6.98	---	*)
TbrPDEB1/TbPDE2C	7.97	---	[20]
TbrPDEB2/TbPDE2B	2.4	---	[21]
HsPDE1A1	112.7	5.0	[38]
HsPDE1B1	24.3	2.7	[38]
HcPDE1C2	1.2	1.1	[38]
HsPDE2	30 – 50	15 – 30	[38]
HsPDE3	0.1–0.8	0.1– 0.8	[38]
HsPDE4	1 – 3	---	[38]
HsPDE5	> 300	1 – 5	[38]
HsPDE6	910	14	[39]
HsPDE7	0.2 – 1	---	[38]
HsPDE8	0.06 – 0.15	124	[38]
HsPDE9	320	0.007–0.17	[38]
HsPDE10	0.05	3	[38]
HsPDE11	0.5	1	[38]

*) this work
